# VIM-2–producing Multidrug-Resistant *Pseudomonas aeruginosa* ST175 Clone, Spain

**DOI:** 10.3201/eid1808.111234

**Published:** 2012-08

**Authors:** Esther Viedma, Carlos Juan, Jennifer Villa, Laura Barrado, M. Ángeles Orellana, Francisca Sanz, Joaquín R. Otero, Antonio Oliver, Fernando Chaves

**Affiliations:** Hospital Universitario 12 de Octubre, Madrid, Spain (E. Viedma, J. Villa, L. Barrado, M.Á. Orellana, F. Sanz, J.R. Otero, F. Chaves);; and Hospital Universitario Son Espases, Palma de Mallorca, Spain (C. Juan, A. Oliver)

**Keywords:** Pseudomonas aeruginosa, bacteria, antimicrobial resistance, multidrug-resistant, outbreak, metallo-β-lactamase, ST175, bacteria, Spain, pandemic, clone, VIM-2 β-lactamase, IMP-22 β-lactamase

## Abstract

A total of 183 patients were colonized or infected with multidrug-resistant *Pseudomonas aeruginosa* isolates at a hospital in Spain during 2007–2010; prevalence increased over this period from 2.8% to 15.3%. To characterize these isolates, we performed molecular epidemiologic and drug resistance analysis. Genotyping showed that 104 (56.8%) isolates belonged to a single major clone (clone B), which was identified by multilocus sequence typing as sequence type (ST) 175. This clone was initially isolated from 5 patients in 2008, and then isolated from 23 patients in 2009 and 76 patients in 2010. PCR analysis of clone B isolates identified the *bla*_VIM-2_ gene in all but 1 isolate, which harbored *bla*_IMP-22_. ST175 isolates were susceptible to only amikacin (75%) and colistin (100%). Emergence of the ST175 clone represents a major health problem because it compromises therapy for treatment of *P. aeruginosa* nosocomial infections.

## Abstract 2

This clone is a major public health problem because it limits antimicrobial drug therapy.

Members of the bacterial genus *Pseudomonas*, especially *P. aeruginosa*, are among the major nosocomial pathogens because of their ubiquitous nature and ability to colonize and survive in hospital reservoirs and because of their role in causing infections in immunocompromised and critically ill patients (*1*). *P. aeruginosa* shows a high level of intrinsic resistance to antimicrobial drugs and an ability to become even more drug resistant. These characteristics are caused by selective pressure of mutations in chromosomal genes that lead to *ampC* hyperexpression, repression or inactivation of *oprD*, and overexpression of efflux pumps (*2*). In addition, *P. aeruginosa* is able to acquire other drug-resistance determinants by horizontal transfer of mobile genetic elements coding for class B carbapenemases (also called metallo-β-lactamases [MBLs]), which hydrolyze all β-lactams except aztreonam (*3*).

Because they can be disseminated horizontally through transfer of resistance determinants, MBLs have become a serious concern in hospitals worldwide over the past decade. Such acquired MBLs include the IMP and VIM types SPM-1, GIM-1, SIM-1, AIM-1, KHM-1, NDM-1, and SID-1 (*4**,**5*). MBL genes are normally encoded in class 1 integrons along with other resistance determinants, such as the aminoglycoside-modifying enzymes. The integrons are frequently located in plasmids or transposons, the dissemination of which contributes to the global spread of this resistance mechanism (*6**,**7*). The versatility and ability of *P. aeruginosa* to combine different resistance mechanisms has led to emergence of strains that are resistant to multiple antimicrobial drugs, which severely limits therapeutic options for treating infections (*8**,**9*). Interim definitions defining multidrug-resistant (MDR), extensively drug-resistant (XDR), and pandrug-resistant bacteria, including *P. aeruginosa*, have been recently reported (*10*).

Although the prevalence of *P. aeruginosa* strains producing carbapenemases in Spain was considered low (0.4% of carbapenem-resistant isolates) (*11*) compared with prevalence in other countries in Europe, such as Italy (12.6% of carbapenem-resistant isolates) (*12*), detection of these isolates is no longer sporadic (*13**,**14*). Recent evidence from multicenter studies indicates an ≈10-fold increase in prevalence of these isolates in the past 5 years (*15*). During 2007 and 2008, a polyclonal outbreak of VIM-2–producing *P. aeruginosa* was detected in a hospital in Spain. At the same time, another outbreak in the hematology department of a hospital in Spain was caused by a *P. aeruginosa* ST235 clone, which produced GES-1 and GES-5, 2 extended-spectrum β-lactamases (*16*). We have observed a sharp increase in infections with drug-resistant *P. aeruginosa* that produces carbapenemase. Thus, we conducted a study to determine the clinical and molecular epidemiologic characteristics of drug-resistant *P. aeruginosa* isolates detected at a major hospital during 2007–2010.

## Materials and Methods

### Study Population

We conducted a retrospective study of all non–cystic fibrosis adult patients who were colonized or infected with *P. aeruginosa* isolates during January 2007–December 2010 at the Hospital Universitario 12 de Octubre in Madrid. This hospital is a 1,300-bed tertiary-care facility serving a population of 600,000 persons (≈42,000 admissions/year). We classified resistance patterns in *P. aeruginosa* according to recently published proposed interim definitions (*10*). An isolate was defined as MDR if it was resistant to >1 drug in >3 categories of drugs and XDR if it was resistant to >1 drug in <2 drug categories. The drugs on which our categorization was based included antipseudomonal cephalosporins (ceftazidime [CAZ], cefepime [FEP]), carbapenems (imipenem [IMP], meropenem [MER]), piperacillin/tazobactam (PIP-TZ), aztreonem, fluoroquinolones (ciprofloxacin [CIP]), and aminoglycosides (gentamicin [GEN], tobramycin [TOB], amikacin [AMK]).

Unique (nonduplicate) clinical isolates from colonized or infected patients were collected during the study. A case was defined as nosocomial if infection or colonization was detected in a person >48 hours after admission or if a person had documented evidence of hospitalization within the previous 12 months. Colonization was defined as isolation of *P. aeruginosa* from >1 clinical specimens in the absence of clinical signs consistent with infection. Medical charts were reviewed and demographic, clinical, and microbiological data were collected.

### Antimicrobial Drug Susceptibility Testing

Identification and antimicrobial drug susceptibility testing of *P. aeruginosa* isolates included in this study were performed by using semi-automated microdilution panels (Soria, Melguizo, Spain) (*17*). The antimicrobial drugs tested were PIP-TZ, CAZ, FEP, ATM, IMP, MER, CIP, GEN, TOB, AMK and colistin. Break points were applied according to Clinical and Laboratory Standards Institute (CLSI) guidelines (*18*).

### Genotyping Analysis

Epidemiologic relatedness of isolates was studied by using pulsed-field gel electrophoresis (PFGE) and multilocus sequence typing (MLST). PFGE was conducted by macrorestriction of chromosomal DNA with *Spe*I and separation of restriction fragments by using a CHEF DRIII PFGE system (Bio-Rad Laboratories, Hercules, CA, USA). Migration of DNA fragments was normalized by using an appropriate mass marker, and computer-assisted analysis of PFGE patterns was conducted by using Bionumerics software (Applied Maths, St-Martens-Latem, Belgium). PFGE types were defined on the basis of DNA banding patterns in accordance with criteria defined by Tenover et al. (*19*).

MLST was performed on selected isolates according to published protocols (*20*). Standard DNA amplification and sequencing of 7 housekeeping genes (*acs*A, *aro*E, *gua*A, *mut*L, *nuo*D, *pps*A, and *trp*E) were performed. Isolates were assigned a sequence type (ST) number according to the allelic profiles available in the MLST database (http://pubmlst.org/paeruginosa).

### Characterization of Acquired MBLs and Integron Analysis

The presence of horizontally acquired β-lactamases was determined by using phenotypic and genetic approaches. Phenotypic tests included analysis with Etest MBL strips (AB Biodisk, Solna, Sweden) for detection of class B carbapenemases. On the basis of positive results from preliminary phenotypic tests, the potential presence of genes encoding acquired metallo-β-lactamases was explored by using PCR amplification and DNA sequence analysis.

Described primers and conditions were used to amplify genes encoding VIM and IMP type β-lactamases (*11**,**13*). After PCR amplification, sequencing reactions were performed by using the BigDye Terminator Kit (PE Applied Biosystems, Foster City, CA, USA), and sequences were analyzed by using an ABI prism 3100 DNA Sequencer (PE Applied Biosystems). Resulting sequences were compared with those available in GenBank (www.ncbi.nih.gov/BLAST). Integrons harboring MBL-encoding genes were characterized by PCR and DNA sequencing by using specific primers to amplify the IntI1 and qacEΔ1 markers, the DNA region located between intI1 and qacEΔ1, and the corresponding MBL-encoding gene (*11**,**13*).

### Statistical Analysis

Univariate analysis was performed by using the *t* test for continuous variables and the χ^2^ or Fisher exact tests for categorical variables. A p value <0.05 was considered significant. Data were stored and analyzed by using SPSS version 17.0 for Windows software (Analytical Software, St. Paul, MN USA).

## Results

### General Characteristics

**Figure 1:**
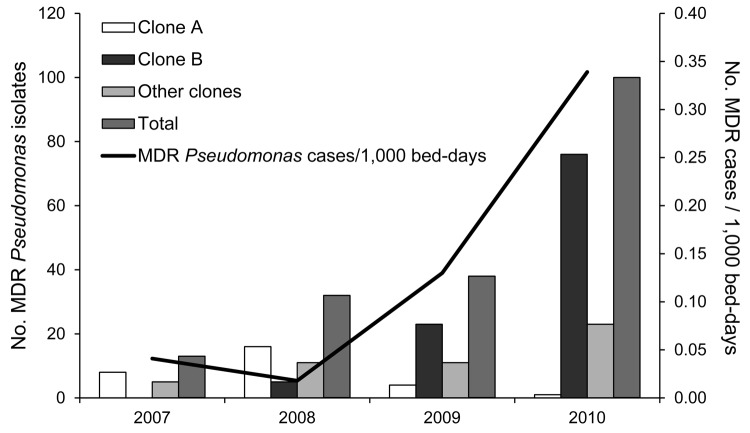
Annual incidence of infections/colonizations by multidrug-resistant (MDR) *Pseudomonas* spp. and temporal distribution of human cases according to clonal type, Spain.

During the 4-year study, of 2,145 patients who were infected or colonized with *Pseudomonas* spp., 183 harbored MDR or XDR isolates: 13 (2.8%) of 460 in 2007, 32 (6.2%) of 517 in 2008, 38 (7.4%) of 514 in 2009, and 100 of (15.3%) 654 in 2010. The estimated annual incidence of MDR *Pseudomonas*–infected persons increased from 0.04/1,000 bed-days in 2007 to 0.34/1,000 bed-days in 2010 (Figure 1). Of 183 isolates, 177 were identified as *P. aeruginosa* and 6 as *P. putida*.

The mean ± SD age of patients was 65.1 ± 15.7 years, and 70.5% were men. Most (95%) cases were nosocomially acquired. The main clinical wards in which drug-resistant bacteria were isolated were internal medicine (57 cases, 31.1%), surgery (32, 17.5%), intensive care (24, 13.1%), pulmonology (21, 11.5%), and hematology (15, (8.2%). Of 183 patients, 143 (78.1%) were considered infected, including 36 (19.7%) with lower respiratory tract infection, 30 (16.4%) with urinary tract infection, 28 (15.3%) with bacteremia, and 22 (12%) with intraabdominal infection. A total of 42 (23%) patients died during hospitalization (Table).

### Molecular Typing of an MDR *P. aeruginosa* Clone

Genotyping analysis of clinical isolates by PFGE showed that 104 isolates belonged to a single major clone (clone B), 29 belonged to a second clone (clone A), and 2 belonged to 2 clones (clones C and D). The remaining isolates, including the 6 *P. putida* isolates, showed unique PFGE patterns.

MLST analysis was performed on 9 isolates in clone B to determine their relationship to other strains that had been described. All isolates that we examined exhibited the same allelic profile (*acsA* [28], *aroE* [22], *guaA* [5], *mutL* [3], *nuoD* [3], *ppsA* [14], and *trpE* [19]), and were identified as ST175 according to the MLST database. MLST analysis of isolates in clone A had already been performed, and these isolates were classified as ST235 (*16*).

Clone B was initially isolated in February 2008 from a patient admitted to the pulmonology ward with a diagnosis of lower tract respiratory infection. Subsequently, 4 additional clone B isolates were detected in 2008, 23 in 2009, and 76 in 2010 (Figure 1). Twelve patients infected or colonized with this clone were referred to Hospital Universitario 12 de Octubre from 4 other hospitals in Madrid and from a fifth hospital in the Canary Islands. From 5 of these patients, the MDR *P*. *aeruginosa* isolate was recovered at the time of admission to Hospital Universitario 12 de Octubre.

Comparison of clinical characteristics for patients infected or colonized with clone B and other MDR *P. aeruginosa* clones did not show major differences, but patients infected with clone B were slightly older (mean age 67.5 vs. 61.9 years; p = 0.016) and they were more frequently admitted to the internal medicine (37.5% vs. 22.8%; p = 0.033) and pulmonology wards (16.3% vs. 5.1%; p = 0.018). Patients infected with clone B had more frequent underlying respiratory disease such as chronic obstructive pulmonary disease (36.5% vs. 22.8%; p = 0.046) (Table) than patients infected with other clones. We also found similar differences between patients infected with clone A and those infected with other MDR *Pseudomonas* spp. clones as reported (*16*).

### Antimicrobial Drug Susceptibility

Antimicrobial drug–resistance patterns of 183 *Pseudomonas* spp. isolates were CAZ (100% resistant), FEP (100%), IMP (100%), MER (100%), GEN (97.3%), TOB (96.7%), and CIP (94%), and resistance to PIP-TZ (50.3%), AMK (42.1%), and ATM (41%) was more variable (Table A1). When break points recommended by the European Committee on Susceptibility Testing (EUCAST; www.eucast.org) were applied, the percentage of PIP-TZ–resistant and ATM-resistant isolates increased to 98.4% and 97.8%, respectively. There were no differences in MIC ranges for the other drugs tested when either CLSI or EUCAST break points were applied. All isolates were susceptible to COL.

Resistance profiles of MDR isolates in clone B were CAZ (100% resistant), FEP (100%), IMP (100%), MER (100%), CIP (100%), GEN (100%), and TOB (100%), and some isolates were also resistant to ATM (13.5%) and AMK (25%). The percentage of isolates resistant to PIP/TZ according to break points recommended by CLSI and EUCAST were 20.2% and 97.1%, respectively (Table A1).

Resistance profiles of isolates in clone A were CAZ (100% resistant), FEP (100%), IMP (100%), MER (100%), CIP (96.5%), GEN (100%), TOB (89.6%), AMK (86.2%) and ATM (86.2%). As a result, this clone was classified as XDR, as were clones C and D (Table A1).

### Detection of MBL Genes

All isolates in clone B were positive by phenotypic methods for MBL. PCR analysis and sequencing identified MBL VIM-2 in 103 isolates and IMP-22 in 1 isolate (Table A1). VIM-2 was also detected in isolates in clone C, in 4 unique clones of *P. aeruginosa*, and in 5 of 6 isolates of *P. putida*. In 1 *P. putida* isolate, VIM-1 and VIM-2 MBLs were detected. A VIM-1-type MBL was also found in 1 clone of *P. aeruginosa* (Table A1). No MBL genes were detected among isolates in clone A, which demonstrated that these isolates were GES-1/GES-5 extended-spectrum β-lactamase/class A carbapenemase producers (*16*). Overall, the percentage of MBL-producing MDR or XDR *Pseudomonas* spp. isolates during the study was 63.9%. Moreover, if one considers the clone producing GES-1-GES-5, the overall prevalence of isolates producing acquired carbapenemases reaches 79.8%.

**Figure 2:**
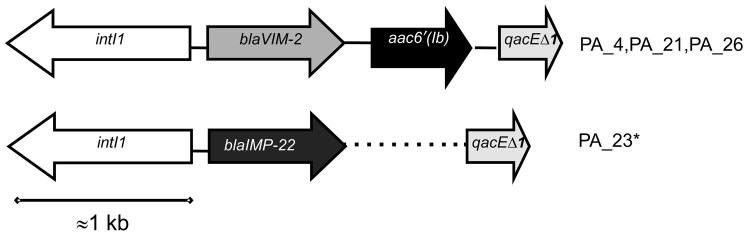
Structure of the class I integron detected in 4 representative isolates of the *Pseudomonas* spp. epidemic multidrug-resistant sequence type 175 clone, Spain. PA, *P. aeruginosa*. *Dotted line indicates undetermined part not amplified by PCR.

To investigate the genetic content of integrons from clone B isolates, we conducted PCR mapping of blaVIM-2 and blaIMP-22 genes for 4 strains. The structure of integrons from isolates producing VIM-2 was IntlI-VIM2-aac6'Ib-qacEΔ1 (Figure 2) in all isolates. IMP-22 was also found to be encoded in a class 1 integron, but we were unable to amplify the fragment between blaIMP-22 and qacEΔ1 by PCR, perhaps because of the large size of this DNA fragment.

## Discussion

Outbreaks by MBL-producing *P. aeruginosa* have been documented in hospitals in several countries, and VIM-is the most dominant MBL variant in Spain and worldwide (*14**,**21**–**27*). We report a large outbreak of VIM-2–producing MDR *P. aeruginosa*. The predominant clone belonged to ST175, a strain that was first detected on February 2008, and it has become increasingly common in Hospital Universitario 12 de Octubre, affecting 104 patients. This MDR *P. aeruginosa* clone was found in in several wards of the hospital, although it was more frequently associated with patients in the internal medicine and pulmonology wards.

The spread of the ST175 clone should be considered an emerging pandemic. It was first identified in 2005 in the United Kingdom and Canada (http://pubmlst.org/paeruginosa) and has been reported in Hungary, the Czech Republic, Poland, Spain, the Unites States, and China (*15**,**28**–**31*). Our study also identified this clone in patients referred to Hospital Universitario 12 de Octubre from 5 other hospitals in Spain, supporting the notion that this clone has disseminated nationwide (*28*). ST175 has been associated with multidrug-resistant isolates and acquisition of different β-lactamases, mostly located on mobile elements such as integrons (*15**,**31**,**32*).

In our study, all MDR *P. aeruginosa* isolates belonging to ST175 producedVIM-2 type MBLs, except for 1 that produced IMP-22. PCR mapping showed that VIM-2 was inserted in a class 1 integron with an aminoglycoside-modifying enzyme (*aac*6′-1b). The IMP-type MBLs are most common among *Pseudomonas* spp. isolates in Asia, although they have been reported less frequently in some countries in Europe such as Italy and Austria (*33**,**34*). We report a lineage of ST175 MDR *P. aeruginosa* that produces IMP-22, which adds this MBL to the list of acquired β-lactamases associated with this epidemic clone. A thorough understanding of the genetic mechanism involved and horizontal and longitudinal dissemination is necessary, particularly for those carrying integron- and plasmid-borne MBLs, given their additional capacity for intraspecies and interspecies spread of multidrug resistance.

Although the origin of the ST175-VIM-2 MDR *P. aeruginosa* strain is unknown, this clone emerged in Hospital Universitario 12 de Octubre in February 2008, seven months after the first detection of VIM-2 type MBLs in a strain of *P. putida*. This species and other *Pseudomonas* species might play a major role as potential reservoirs for MDR determinants by enhancing their transfer to *P. aeruginosa* clones (*3*).

The ST175 clone was able to persist in Hospital Universitario 12 de Octubre for >34 months and has disseminated widely in spite of control measures that have been implemented, such as strict isolation of patients, active surveillance of patients at the time of entry into intensive care units, and environment investigation of possible sources of colonization. The spread of this clone among patients admitted to different sections of the hospital and high selective pressure for antimicrobial drug resistance may encourage its persistence. The design and implementation of infection control strategies in these hyperendemic situations is challenging. We recently faced a similar situation in Hospital Universitario 12 de Octubre with a large outbreak of MDR *Acinetobacter baumannii* that persisted for >30 months but that was finally controlled (*35*).

This study also detected emergence of multiple strains of *Pseudomonas* spp. that produced VIM-2- and VIM-1-type MBLs, including >6 *P. aeruginosa* and 6 *P. putida* clones. The polyclonal nature of MBL-based resistance might have major epidemiologic implications because sporadically isolated strains may eventually spread in the hospital environment or act as a reservoir for horizontal transfer of resistance determinants.

The emergence of MBL-producing MDR *P. aeruginosa* is a major health problem because it leaves the clinician with almost no therapeutic options for treating nosocomial infections caused by *P. aeruginosa*. Our results showed that isolates belonging to ST175 had susceptibility only to PIP/TZ (79.8%), ATM (86.5%), AK (75%), and colistin (100%). The difference in PIP/TZ susceptibility depending on the break point criteria applied is remarkable. When the EUCAST susceptibility testing criteria were applied, only 2.9% isolates were susceptible to PIP/TZ compared with ≈80% when criteria recommended by CLSI were applied. A recent report concluded that in *P. aeruginosa* bacteremia caused by isolates with reduced PIP/TZ susceptibility (32/4 µg/mL or 64/4 µg/mL), empirically prescribed PIP/TZ therapy was associated with increased patient deaths (*36*). In our study, most isolates had PIP/TZ susceptibility in this range. Fortunately, the 2012 CLSI PIP/TZ break point (*37*), which was implemented during the review of this report, has been reported as 16/4 µg/mL, thus agreeing with the break point established by EUCAST. Confluence of susceptibility testing criteria among agency standards are useful for optimizing strategies to treat severe MDR *P. aeruginosa* infections.

In summary, we report a large outbreak of infections caused by a VIM-2–producing ST175 MDR *P.* *aeruginosa* strain that was responsible for 76% of infections or colonizations by MDR *P*. *aeruginosa* in 2010 at Hospital Universitario 12 de Octubre, and >50% of infections or colonizations during the study period. This epidemic clone is also circulating in other hospitals in Spain and other countries. The underlying reasons for the widespread success of this clone still need to be fully elucidated fully, including the potential for an enhanced ability to acquire MDR determinants that facilitate persistence under conditions of antimicrobial drug selective pressure encountered in the hospital environment (*21**,**22*). Deciphering the epidemiologic and molecular aspects driving the emergence and spread of such strains is crucial to the implementation of efficient measures to control their dissemination.

## Appendix

**Table A1:** Clonal distribution, type of carbapenemase, and drug resistance patterns of MDR *Pseudomonas* isolates, Spain, January 2007–December 2010 *

Isolate	No. isolates/y					Carbapenemase type (no. isolates)	ST	No. (%) resistant isolates									
	2007	2008	2009	2010				PIP/TZ	CAZ	FEP	IMP	MER	ATM	GEN	TOB	AMK	CIP
All MDR isolates (183)	13	32	38	100		VIM-1, VIM-2, IMP-22, GES1–5 (146)	NA	92 (50.3)/ 180 (98.4)	183 (100)	183 (100)	183 (100)	183 (100)	75 (41.0)/ 179 (97.8)†	178 (97.3)	177 (96.7)	77 (42.1)	172 (94)
*P. aeruginosa* (clonal types)																	
B (104)	0	5	23	76		VIM-2 (103), IMP-22 (1)	175, 175	21 (20.2)/ 101 (97.1)†	104 (100)	104 (100)	104 (100)	104 (100)	14 (13.5)/ 100 (95.2)†	104 (100)	104 (100)	26 (25)	104 (100)
A (29)‡	8	16	4	1		GES 1/5 (29)	235	29 (100)	29 (100)	29 (100)	29 (100)	29 (100)	25 (86.2)	29 (100)	26 (89.6)	25 (86.2)	28 (96.5)
C (2)	0	0	1	1		VIM-2 (2)	NA	2 (100)	2 (100)	2 (100)	2 (100)	2 (100)	2 (100)	2 (100)	2 (100)	2 (100)	2 (100)
D (2)	0	0	0	2		Noncarbapenemase (2)	NA	2 (100)	2 (100)	2 (100)	2 (100)	2 (100)	0	2 (100)	2 (100)	2 (100)	2 (100)
Unique clones (40)	4	10	8	18		Noncarbapenemase (35)	NA	31 (88.6)/ 35 (100)†	35 (100)	35 (100)	35 (100)	35 (100)	26 (74.3)	30 (85.7)	32 (65.7)	17 (48.3)	25 (71.4)
	0	1	0	0		PA_5 VIM-2	NA	0	0	0	0	0	0	0	0	0	0
	0	1		0		PA_10 VIM-2	NA	0					0	0	0	0	0
	0	0	1	0		PA_12 VIM-1	NA	4 (80)/ 5 (100)†	5 (100)	5 (100)	5 (100)	5 (100)	4 (80)	5 (100)	5 (100)	3 (60)	5 (100)
	0	0	1	0		PA_13 VIM-2	NA	0	0	0	0	0	0	0	0	0	0
	0	0	0	1		PA_28 VIM-2	NA	0	0	0	0	0	0	0	0	0	0
*P. putida* (clonal types)																	
Unique clones (6)	1	1	2	2		0	NA	0	0	0	0	0	0	0	0	0	0
	1	0	0	0		PP_1 VIM-2	NA	0	0	0	0	0	0	0	0	0	0
	0	1	0	0		PP_11 VIM-2	NA	0	0	0	0	0	0	0	0	0	0
	0	0	1	0		PP_14 VIM-2	NA	3 (50)/ 6 (100)†	6 (100)	6 (100)	6 (100)	6 (100)	4 (66.6)	6 (100)	6 (100)	2 (33.3)	6 (100)
	0	0	1	0		PP_19 VIM-2	NA	0	0	0	0	0	0	0	0	0	0
	0	0	1	0		PP25_VIM-1/VIM-2	NA	0	0	0	0	0	0	0	0	0	0
	0	0	1	0		PP_27 VIM-2	NA	0	0	0	0	0	0	0	0	0	0

*MDR, multidrug resistant; ST, sequence type; PIP/TZ, piperacillin-tazobactam; CAZ, ceftazidime; FEP, cefepime; IMP, imipenem; MER, meropenem; ATM, aztreonam; GEN, gentamicin; TOB, tobramycin; AMK, amikacin; CIP, ciprofloxacin; NA, not available. None of the isolates showed resistance to colistin. †Percentage drug-resistant isolates when the European Committee on Susceptibility Testing break point was applied. ‡Viedma et al. (*16*).

**Table:** Characteristics of 183 patients colonized or infected with clone B or nonclone B multidrug-resistant *Pseudomonas* isolates, Spain, January 2007–December 2010*

Characteristic	Patients with clone B, n = 104	Patients with non–clone B, n = 79	p value
Mean ± SD age, y	67.5 ± 15.4	61.9 ± 15.7	0.016
Male	74 (71.1)	55 (69.6)	0.822
Hospital location			
Surgical ward	15 (14.4)	17 (21.5)	0.211
Intensive care unit	11 (10.6)	13 (16.4)	0.243
Internal medicine ward	39 (37.5)	18 (22.8)	0.033
Hematology ward	4 (3.8)	11 (13.9)	0.014
Pulmonology ward	17 (16.3)	4 (5.1)	0.018
Hospitalization within previous 3 mo	48 (46.1)	37 (46.8)	0.927
Surgical procedure	29 (27.9)	32 (40.5)	0.073
Transplant	4 (3.8)	8 (10.1)	0.089
Hematologic malignancy	8 (7.7)	11(13.9)	0.171
Solid tumor	21 (20.2)	13 (16.4)	0.520
Diabetes mellitus	29 (27.9)	21 (26.9)	0.845
Chronic obstructive pulmonary disease	38 (36.5)	18 (22.8)	0.046
Central catheter	83 (79.8)	59 (76.7)	0.410
Urinary catheter	60(57.7)	44(55.7)	0.441
Infections	82 (78.8)	61 (77.2)	0.791
Respiratory tract	25 (24)	11 (13.9)	0.088
Intraabdominal	7 (6.7)	15 (19)	0.012
Wound	4 (3.8)	8 (10.1)	0.089
Urinary tract	19 (18.3)	11 (13.9)	0.432
Bacteremia	12 (11.5)	16 (20.2)	0.105
Died during hospitalization	20 (19.2)	22 (27.8)	0.170

*Values are no. (%) unless otherwise indicated.
